# Syntax of Testimony: Indexical Objects, Syntax, and Language or How to Tell a Story Without Words

**DOI:** 10.3389/fpsyg.2019.00477

**Published:** 2019-03-22

**Authors:** Till Nikolaus von Heiseler

**Affiliations:** Institute of Philosophy, Freie Universität Berlin, Berlin, Germany

**Keywords:** arbitrarisation, index, language evolution, sign-language, storytelling, testimony, trophy, Peircean linguistics

## Abstract

Language—often said to set human beings apart from other animals—has resisted explanation in terms of evolution. Language has—among others—two fundamental and distinctive features: syntax and the ability to express non-present actions and events. We suggest that the relation between this representation (of non-present action) and syntax can be analyzed as a relation between a function and a structure to fulfill this function. The strategy of the paper is to ask if there is any evidence of pre-linguistic communication that fulfills the function of communicating an absent action. We identify a structural similarity between understanding indexes of past actions of conspecifics (who did what to whom) and one of the simplest and most paradigmatic linguistic syntactic patterns – that of the simple transitive sentence. When a human being infers past events from an index (i.e., a trace, the conditions of a conspecifics or an animal, a constellation or an object) the interpreters’ comprehension must rely on concepts similar in structure and function to the ‘thematic roles’ believed to underpin the comprehension of linguistic syntax: in his or her mind the idea of a past action or event emerges along with thematic role-like concepts; in the case of the presentation of, e.g., a hunting trophy, the presenter could be understood to be an *agent* (subject) and the trophy a *patient* (direct object), while the past action *killed* is implied by the condition of the object and its possession by the presenter. We discuss whether both the presentation of a trophy and linguistic syntax might have emerged independently while having the same function (to represent a past action) or whether the presentation of an index of a deed could constitute a precursor of language. Both possibilities shed new light on early, and maybe first, language use.

## Introduction

Language makes us human. It is the basis of cumulative culture, technology, and perhaps our exceptional success as a species. At the same time, the problem of language evolution has been considered one of the hardest problems in science today (Christiansen and Kirby, 2003). According to the Australian science journalist Christine Kenneally (2008), scientists from all relevant fields—including linguists, geneticists, philosophers, neuroscientists, anthropologists, and psychologists—have engaged in “a cross-discipline, multidimensional treasure-hunt” for the mechanisms of language evolution.

Over the past 30 years, during which the origins of language have been studied with increasing intensity, there have been, according to Hauser et al. (2002), three theoretical issues cross-cutting the debate. The first and one of the oldest scholarly disagreements about language evolution concerns whether it is discretely—non-gradually— distinguishable from animal communication. Today most scientists agree that “although bees dance, birds sing, and chimpanzees grunt, these systems of communication differ qualitatively from human language” (Hauser et al., 2002).

The second issue structuring the debate has been whether this distinction also implies a discontinuity in the evolutionary history of language. Evidence supporting the essential difference between human language and animal communication includes that they are processed in very different brain regions (Arbib, 2002). Instead of animal communication, the action-recognition system of primates—composed of *mirror neurons*—has been suggested as a basis for the development of the language faculty, and for good reason: both the mirror neuron system and Broca’s area (a human brain region linked to speech production) are located in the frontal lobe of the left hemisphere (Arbib, 2005). The neurological relatedness of the mirror neuron system (or any other yet undiscovered brain area necessary for action recognition) with language (Rizzolatti and Sinigaglia, 2008) makes sense functionally; building any sentence depends on recognizing the conceptual categories of actions (or states or events) expressed by verbs.

The third issue—crystallizing the current debate—is whether language emerged gradually over millions of years through the extension of preexisting communication systems or whether important, highly specialized features that developed for other reasons were at one point integrated, making a sudden development possible.

Besides these three issues (and related to the third), there has been an intensive debate about whether any principles of cooperation and pragmatic cognition must have been active in human interactions before linguistic behavior could begin, some researchers even suggesting some kind of altruistic behavior—a distinctive form of cooperation—as the foundation of language evolution (e.g., Tomasello, 2008). Other researchers explain anomalous human altruism as an outgrowth of language use (Nowak and Sigmund, 2005), such as, specifically, the ability to report the past behavior of individuals (von Heiseler, 2015).

## Structure and Function

If we accept the premise that language evolved for communication (rather than for thinking), the take-off point for any discussion on the origin of language is the array of features uniquely setting language apart from other forms of communication. A tentative assumption of our proposal is that there are—among others—two fundamental and distinctive features of language:

(1) Syntax: Traditionally, the capacity of language *to express an infinite number of propositions using finite means* (productivity) was regarded as its most unique quality (e.g., Chomsky, 1991). However, the simplest syntactic structures do not manifest this property as much as they do the capacity of syntax to represent the relations between entities in events through argument structure.
(2) Storytelling: It has been supposed that another unique feature of language is *its ability to represent non-present events, actions, and states.* In the last decade, the importance of narratives has been broadly accepted in cognitive science (Turner, 1996; Bloom, 2005) and it has been proposed that narration might have played a crucial role in language evolution (e.g., von Heiseler, 2014; Corballis, 2014, 2015; Ferretti et al., 2017).

We suggest that the relation between the ability to present non-present events and syntax can be analyzed as a relation between a function and a structure to fulfill this function. In human language, words are used in syntactic structures (Chomsky, 1965) expressing thoughts (Jackendoff, 2002), which most simply and typically represent past, future, or imaginary actions (Corballis, 2016a,b) or as Pinker (2007, p. 45) puts it: language gives “us a way to communicate *who did what to whom*.”

While animal communication consists—for the most part—of isolated signals interpreted (or more accurately, reacted to) each in their own right, linguistic utterances can only be understood when multiple elements are interrelated: minimal syntax – i.e., argument structure – seems a necessary condition for referring to past events. The unique structure of language (syntax) and its unique function (storytelling) therefore seems closely inter-related. More evidence that language is adapted to the function of relating events—and especially human actions—is its complex temporal logic and the fact that verbs (most of which refer to actions) dictate the syntax of sentences (Jackendoff, 1983) by dictating their thematic structure and by inter-relating thematic roles (such as agent, patient, and instrument). In other words, the assignment of entities to conceptual (semantic) thematic roles is an essential function and cognitive pre-condition of even the simplest forms of syntax.^1^ Although most linguists seem to agree that the meanings of thematic roles may vary substantially between contexts, it has also been noted that the most basic thematic role distinction—the distinction between *agent* and *patient*—is most clearly pronounced in the use of the verb “to kill” (Dowty, 1991). Furthermore, it has been argued that a grammatical construction inter-relating thematic roles can be interpreted as a basic narrative structure—that “the abstract narrative structure is projected to create the abstract grammatical structure” (Turner, 1996, p. 143). This leads us to the working hypothesis of this paper: syntax can be conceptualized as structure adapted to the function of *communicating who did what to whom.*

Accepting this perspective—and the fact that in evolution, structure follows (within constructional restrictions and the constraints of evolutionary history) function—we might imagine that *something* would be used to refer to absent actions and that *this* would develop into language. This implies that the ability to refer to past events must, under some circumstances, bequeath a reproductive advantage, such that the structure of language would evolve as an adaptation to this function.

Non-human animals lack two essential skills: first, the syntactic ability: they seem to decode each signal instantly and by itself. Secondly, their ability to understand *mimetic gestures* seems very limited. Before we look more deeply into the problem of mimesis and the requirements for understanding mimetic gestures, we need to address the syntactic ability and its essentiality to language.

## What We Can Learn From Improvised Sign Language

The semantics and structure of simple syntax is especially easy to illustrate using sign language (although the syntax of sign language is not necessarily less complex than that of spoken language), because its syntactic structure is sometimes visible in space. If I sign in American sign language (ASL) the sentence “I tell you, I will come tomorrow” (meaning, “I promise you I will come tomorrow”) then the *direction* of the main verb, “tell,” marks the subject (I) and the indirect object (you). “I tell you” is one sign, *directed* from the sender (I) to the receiver (you), marking the former as *agent* and the latter as *recipient, goal*, or *patient* (depending on one’s theoretical commitments). There is no need to symbolize the *agent* and the *patient* beyond directing the verb. Because the verb “to tell” is trivalent (creating three slots—the *teller*, the *receiver*, and what is *told*) we cannot understand the proposition until these three pieces of information (teller, receiver, and what is told) are understood. This is to say, the sentence “I tell you” is incomplete *in a very specific way*: it produces the question, ‘what are you telling me?’ Thus, the receiver searches for anything—indicated or implied—that *could be told.* In this case, she or he will find the phrase “I come tomorrow.” If the receiver does not find the necessary information, he or she must guess or request clarification. Most significantly for us, a single *directed mimetic gesture* can associate *present objects* (e.g., the sender and the receiver) with thematic roles (e.g., *agent* and *patient*) and potentially represent a non-present action or event. While it is generally impossible to express a non-present action with a single ‘word’ or morpheme (conjugated verbs of pro-drop language as must consist of at least two morphemes, the stem and the inflectional morpheme), a gesture (e.g., the *mimetic* sign of the verb) can do so because it has the potential to integrate present objects (by marking them for thematic roles), for instance by its direction.

In the theaters of our imaginations, we shall now stage a classic scene—boy meets girl—in a kind of improvised sign language: say you are the boy and I am the girl. You come up to me and point alternately to yourself and to me; then you shape the right hand as if holding a cup and bring this imaginary cup to your mouth. Finally, you point in one direction and then point again to yourself and me, to us. How should I interpret these signs? Any human would understand that it might mean something like, “Would you like to have a coffee with me?” Let us now analyze how this meaning is constructed, there being an *explicit proposition*, consisting of signs in a syntactic structure, and *pragmatic implications*.

First, the explicit part: you made three gestures: (1) the alternating pointing at you and me; (2) the mimetic gesture of drinking from a cup; and (3) a gesture pointing in one direction. My innate and universally human ability to understand syntax-like constructions (here the argument structure) is demonstrated by the fact that I expect such a structure and comprehend instinctively these three signs as a unity. Without this syntactic expectation, we would not understand the meaning of the sentence. We would not even understand that we did not understand.

Now, let us go a step further and analyze the used signs one by one and how they manage to connect to each other. The pointing to you and me means *you and I* or *we* (in no specified grammatical case or thematic relation). In this improvised signing there is (in contrast to conventional sign languages) no difference between ‘you and I’ and ‘we.’ Now the receiver works to a certain extent like a Turing machine; she gets two items of information—the *semantic meaning* of the sign (e.g., word) and the *go-to-command* consisting of a *search order* saying ‘go to the missing syntactic element that is needed for a minimal well-formed syntactic structure.’

In this case, the ‘we’ will be interpreted as the agent and the search command is ‘look for a verb that could relate to this agent.’ This is the mimetic gesture, ‘drinking coffee.’ Now we have a complete sentence: *We drink coffee.* From this another search command emerges. This search surpasses syntactic demands, because the sentence is grammatically complete. Here, we enter the world of pragmatics. But before we do so, let us reconstruct how the simple sentence “We drink coffee” is built. It is built out of two elements: ‘we’ and ‘to drink coffee’ (the latter expressed by one sign). ‘We’ is a dynamic indexical gesture and ‘to drink coffee’ is the mimetic gesture of drinking from a cup with handle. To understand the sentence, I must *replace ‘*you’ (the ‘I’ that was in implied in your gesture, because *you* were pantomiming drinking from a cup) with ‘we.’ The *search* command and *replacement* seem to be basic operations of our syntactic faculty. They work together; first, every slot needs to be filled, but even if we already understand a complete sentence, we can always replace elements when new information comes in, as we shall see later.

As said, the sentence ‘We drink coffee’ is complete. However, I—the receiver of the completed sentence—ask myself, when and where? This is a new search command also concerning the *pragmatic meaning.* Pragmatics includes knowledge of the world and puts the utterance in context. Obviously, we are not drinking coffee in the present. Also, I know that we have never drunk coffee together before. Because the event of drinking coffee was not in the past and is not happening in the present, I assume you are talking about a possible future. So, I (in the scenario: the girl, the receiver) suppose that the context will tell me where and when this action should happen. This is to say that the tense of the proposition is implied and that I need to exclude all possibilities but one (performing a *disjunctive syllogism*) to understand that implication. Now I search for the *where*. What I find is the indexical sign for the coffee shop. Instead of pointing there, you could sign another mimetic gesture that would convey the information, or you could show a picture of a coffee shop on your phone. On this first level of pragmatics, the implied sentence is this: ‘We will have coffee in that coffee shop over there.’ But there is a second level of pragmatics concerning how to make sense of the whole situation and the function of the utterance within it. This includes the fact that you are a boy and I am a girl, that we do not know each other and just met for the first time, and knowledge about the conventions of the society we live in. On that level, I, the girl, would interpret the utterance as a polite request or suggestion, something like the question ‘Do you want to have coffee with me in the coffee shop over there?’ in the broader context of a possible friendship or flirt.

Now, what is special about language? On the most complex level, where all information gets integrated into the pragmatics of everyday life, there seem to be some similarities between human and non-human animals. Dogs for instance understand wooing. They have different rituals of courtship, but similar intentions. Non-human animals can also interpret signals in context. Furthermore, the pointing at the coffee shop as a suggestion to go there is within the range of dog comprehension [in contrast to chimpanzees, who do not understand pointing under natural conditions (Kaminski et al., 2009)]. However, the pointing ’me-you-me-you-me-you’ would confuse them and probably be understood as an invitation to play. There seems to be no evidence that non-human, untrained animals can integrate the signs ‘you’ and ‘I’ into ‘we.’

Besides what we like to call the *search algorithm of grammar, replacement* (in our case, of the agent) seems a central element of grammar: I (the girl) saw you making a mimetic gesture *drinking from a cup* and I replaced the original agent *you* (the boy) with the previously signed gesture *I-you-I-you-I-you* meaning ‘we.’ If you now add another gesture to specify a drink—for example by pantomiming opening a bottle of champagne—this champagne will replace the coffee. In this case, we reinterpret the already interpreted signifiers. Either this would be interpreted as a further suggestion or the first mimetic gesture would be interpreted more generally as ‘Let’s go for a drink’ and the new sign would specify the drink.

Now, to pursue the argument further, we need to consider the cognitive complexity of interpreting syntax. Language is processed on multiple levels *simultaneously*. The understood proposition is not written in stone but ready for modification. The process of modification includes at least three levels: (1) the level of semiotics, (2) the syntactic level (connecting semantic elements and allotting to them thematic roles), and (3) the two levels of pragmatics. Each level can influence the interpretation of any other level: when signals do not fit into the pragmatic or syntactic structure, they get reinterpreted on the semiotic level (especially, e.g., homophones). It is even possible that the whole situation gets reinterpreted. Sometimes, likewise, the thematic roles change in the process of understanding. An example: “Noam is too angry to call.” is ambiguous. The verb “to call” produces two slots, the caller and the person called. Now compare the two ideas: *Noam is too angry to make a phone call* versus *we should not call Noam because he is so angry*. These are completely different scenarios. The listener must wait for contextual information and if her assumption changes, she has to reinterpret the utterance. The vast processing costs of language result partially from this iterative reinterpretation. Often the proper construction can only be found by excluding other possibilities. For example, if we interpret the sentence “Noam is too angry to eat,” we normally exclude the possibility that Noam can be eaten—(cf., Chomsky, 2007)—and thus interpret the sentence something like ‘Noam will not eat anything, because he is so angry.’

The analysis of these three levels has different functions for our research: while most parts of the pragmatic level are not specific to language (some aspects must be older than language and others built upon it—as shown in our scenario)—the other two levels offer valuable information concerning language evolution. Non-human animals lack two essential skills: first, the ability to search for missing elements and put them together into a syntactic structure; in contrast, they seem to decode each signal instantly and by itself. Second, they have difficulties to understand mimetic gestures such as ‘drinking from a cup.’

While the syntactic level has long been the center of attention (mostly in much more complex forms than we can discuss in this paper), the semiotic level has been a blind spot in the discussion of language evolution so far. In this section, we discussed how simple language works; the next section brings us to the key question of this research: how is *syntactic competence* related to the uniquely human faculty for *symbol use*, including the ability to produce and understand mimetic gestures.

## Peirce’s Index, Icon, and Symbol

Peirce (1931), developed a complex semiotic theory, of which only a small portion became well-known outside his field of study. This is his classification of signs according to the way they denote objects:

(1) The *index* is a sign that points to its object or relates to it logically. An indexical sign can be either a pointing finger or such as smoke for fire.
(2) The *icon* connects the signifier and the signified via similarity. An iconic sign could be a figurative object or a mimetic gesture.
(3) The *symbol* relates the signifier and the signified by contingent convention; it must be learned; for instance: letters, words, and technical codes.

**FIGURE 1:**
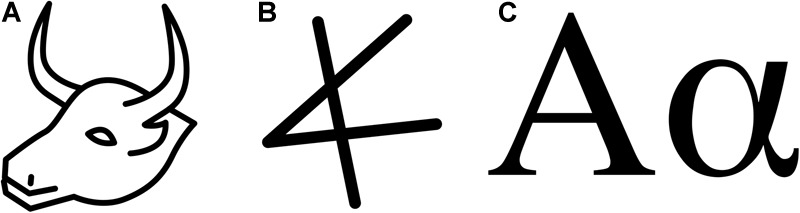
Arbitrarisation. **(A)** Egyptian hieroglyph representing a bull-head, **(B)** Phoenician glottal stop [?], first letter of the Phoenician alphabet **(C)** The first letter of the Greek alphabet: Alpha [a] – (as a capital and as a small letter).

In some textbook representation of Pierce’s theory, the index, the icon, and the symbol are treated as three categories of a similar value. However, there is an essential discrepancy; while the distinction between an icon and a symbol is *gradual*, the difference between icons and symbols on one hand, and an index on the other, is *discrete*. This has essential consequences for a potential evolutionary theory of language; *icons can undergo gradual development through use.* Thus, it is easy to construct an evolutionary story of increasing abstraction. A well-known example is the development of *Nicaraguan sign language* (NSL) from mostly mimetic (Goldin-Meadow and Mylander, 1998) home-signs into a conventional full-fledged language (Senghas et al., 2004). Another example would be the development of the first letter of our alphabet from the Egyptian hieroglyph for *ox head* through the Phoenician *Aleph* (see Figure 1).

This gradual transformation from icon (having features of similarity) to symbol (conventional signs) we refer to as *arbitrarisation*—the transformation of the elements of a communication system toward communicative efficiency—which is to communicate as much as possible as briefly as possible, while minimizing the danger of confusion. By the end of the process each sign is shaped by communicative efficiency; signs, while conditioned by historical and technical constraints, tend to find their places in the virtual space of maximal difference from all (or significant) other signs.^2^ In the end of this process—driven by the selection of efficiency, only *the differences between signals* are finally encoded. As a necessary side-effect, the analogy to the signified object vanishes.

This process of *arbitrarisation* is completely distinct from the ability of signs to take part in syntactic structures: non-language signal systems can undergo the process of *arbitrarisation* and *mimetic* and *conventional signs* can be functional equivalents within a syntactic structure.^3^ This can be clarified by cross-cutting the two properties (see Table 1).

**Table 1:** | Semiotic independency.

	Index	Syntactic symbol
No similarity between signified and signifier	Smoke for fire	Words
Mimetic sign (Icon)	The shape of a shadow (and any technical imaging)	Sign for “house” in American sign language (ASL)

The arbitrariness of signals should not to be confused with their ability to take part in syntactic structures. This can also be demonstrated by the alarm calls of vervet monkeys, which use different signals for different dangers (Seyfarth et al., 1980). Though it would be technically correct to speak of these calls as “arbitrary” it may be misleading; especially in contexts where arbitrariness would be associated with language. For the same reason, the development of Nicaraguan sign language (or any other language) cannot be a model for the emergence of the language faculty, but only a model for *arbitrarisation.*

At this point we need to adapt our terminology to its subject: the category *symbol* is opposed to *index* and divided into *mimetic symbol* (Peirce’s icon) and *arbitrary symbol* (Peirce’s *symbol*). Another argument that mimetic and arbitrary symbols are not discretely distinguishable is that some signs in sign language are conventional and mimetic at the same time; you cannot understand them without learning them, but having learned them, you understand the mimetic relation between signifier and signified. In a nutshell: the difference between arbitrary signs (Peirce’ icons) and conventional signs (Peirce’ symbols) is only gradual, while the distinction between the two on one hand and indexes on the other is fundamental. From this perspective the emergence of language must include the transcendence of the essential distinction between the world of indexes and the cosmos of mimetic or arbitrary symbols.

The idea presented in this paper is based on two premises: (1) evolution adapts whatever already exists for new purposes, (2) While not all indexes are of natural meaning, *all signs with natural meaning can be interpreted as indexical.* The hypothesis that language evolution takes off from indexes is suggested by these premises: the first premise suggests that non-natural meaning (cf. Grice, 1957) developed from natural meaning and from the second premise we can deduce that non-natural meaning means indexical.

Indexes are closely related to the world itself, up to a point, that sometimes the line between objects and indexes of an objects blur. Is the ear of a donkey behind a bush an index of the donkey or the donkey itself? In a more radical view, the world presents itself to us in the form of indexes, because all of our sense perception could be called indexical.

Symbols can refer to displaced (future, past, imaginary) actions through minimal syntactic structures, while the ability of indexes to refer to displaced actions is inflexible and limited. While an indexical finger (pointing) only can refer to present objects or events, indexical objects – as we will see – sometimes can refer to past actions or events. In the model presented here we suggest a transformation from a natural index to an index presentation. In the next passage we will investigate the differences between the world of indexes and language more closely.

## Three Aspects of the Uniqueness of Language

While non-human vertebrates (including non-human primates) live—roughly speaking—in a world of indexes (which in most cases refer to the here and now), people also live in a symbolic world (including mental time traveling and the inner presentation of non-present entities of all sorts, including significant others and persons of reference). Most researchers agree that mental time traveling is limited or non-existent in non-human animals (Suddendorf, 2013). The gap between indexes and symbols can be investigated in terms of their different functions and in terms of the cognitive requirements for the comprehension of mimetic and symbolic signs and operations. While an index, outside of human language, generally stands alone as a mere indicator *that something is the case* (including something’s existence), mimetic or arbitrary symbols are necessary for syntactic structures; no grammatical sentence can be constructed out of indexes alone.

The abilities of non-human animals to understand spontaneously new mimetic symbols, such as pantomime and mimetic objects, are limited to non-existent. Great apes have been conditioned to use some gestures from conventional sign language, but there is no evidence that they can spontaneously understand new mimetic symbols. They also find it difficult to imitate gestures without objects (Tennie et al., 2012). In contrast, people not only comprehend mimetic symbols, but seem to have an innate attraction to mimesis (play) and mimetic objects (such as symbolic toys and puppets).

Further evidence of this difference between human beings and other primates lies in a known correlation between the comprehension of actions (or lack thereof) and the behavior of mirror-neurons: non-human primates seem to understand (categorize) only “transitive actions” (Rizzolatti and Craighero, 2004): actions involving objects or other animals. Their mirror-neurons do not fire when they observe non-transitive actions (such as *walking* or *dancing*—actions of an agent not involving another animal or an object) (Luppino and Rizzolatti, 2001). In the case of human beings, the nervous system reacts not only to non-transitive actions (Maeda et al., 2002), but also to simulations of actions such as picking up an imaginary cup or any other pantomime (Buccino et al., 2001;Grèzes et al., 2003).

The uniqueness of language includes at least three aspects: (1) syntax (in its most basic form: the argument structure: the verb valency with thematic roles), (2) on the level of the function, the representation of non-present (such as past or future) events or actions, and (3) at the semiotic level, the overcoming of the discontinuity between index and mimetic symbol.

Any explanation for language evolution needs to account for how these discontinuities could have been overcome. The classical generative theories concerned mainly the emergence of syntax (e.g., Chomsky, Pinker, Jackendoff); during the last decades, there have been several suggestions regarding the evolution of storytelling (e.g., Dunbar, Corballis, von Heiseler); however, the unique semiotic aspect of language evolution has been widely overlooked.

## Reproductive Advantage Could Have Preceded the Intention to be Understood

We have argued that one of the most unique and central functions of language is the representation of absent actions and that this function depends on syntax-like conceptual structures and (mimetic or arbitrary) symbols. Why is it—in most cases—impossible to refer to an absent action using only indexes? As we saw in the boy-meets-girl scenario, it is possible (if the agents of the related action are present) to refer to the agents by pointing. What about the verb? There are at least two ways to refer to an action by pointing: pointing at a present action or at an object that implies an action. However, this would require that the action in question occur in the presence of the communicative partners or that an object that implies the action is present. Language requires referring to actions without these strict limitations. The simplest way to refer to any absent action is to imitate that action. Such imitation may be done without objects that would be involved in the real action. In sign language, such signs are called *manipulators* (in contrast to *substitutors*). *Manipulators* imitate an action (for example drinking from a cup), while *substitutors* imitate an object or a part of it. For instance, the sign for champagne can combine a *manipulator* (opening the bottle) and a *substitutor* (the effervescent champagne squiring out of the bottle). The benefit of mimetic signs is that they can refer without presupposing any semiotic conventions.

Thus, we have claimed that representing absent actions requires syntax-like structure and mimetic or arbitrary symbols. This urgently compels the question of how and why such activity could have begun; what sort of interactions could form the basis for an adaptation that would evolve into the ability to communicate an absent action, by using a mimetic symbol (of this action) within a minimal argument structure.

A natural place to look for this bridge would be among natural indexical signs, for several reasons; (1) they occur automatically in a natural environment; (2) they are likely to be related to food gathering, hunting and territorial conflicts (e.g., animal tracks, animal homes, feces, evidences of activities of conspecifics such as chipped stones and broken branches, or dangers (of fire and flood for instance) making their interpretation subject to natural selection; interpreting such signs could spell the difference between reproductive success and otherwise; and (3) our ancestors could have responded instinctively to some such natural signs before the ability to interpret them consciously evolved.

In general, as stated above, indexes refer to *present* objects, actions or events. Nevertheless, there are exceptions: for instance, traces left by an animal’s passage can indicate (for those that can read them) that an animal of a certain kind recently performed some action (such as walking or scratching its back on a tree).

In such a case, even if the interpreter imagines a past event (implying concepts of actions, agents, and perhaps other thematic roles) there seems no way that reading traces could develop into something language-like; because unlike interpreting language, reading an animal’s trace lacks the potential for reciprocal communication; the creator of the index (e.g., the animal the scratched its back) is not present and in most or all cases has no intention to communicate.

However, there exists a class of situations in which the receiver can infer a past action of a *present* individual; for instance, if a dominant individual shows signs of repletion while another individual gulps down some flesh it bites off from a bone, the observer can infer that the dominant individual gave the bone to the other individual after eating to satisfaction. Also, in this case there is no intention to communicate. Another example: an observer might infer that the individual carrying a hand ax produced it himself.

In both cases, the past of the observed individual might be imagined, and thus a non-present event conceptually represented; thereby valuable information about the social world could be transmitted. We know that primate societies depend on a variety of sophisticated cognitive skills, and it has been claimed that such abilities evolved in response to social demands (Humphrey, 1976) and action reasoning (Fabbri-Destro and Rizzolatti, 2008). It seems likely that being able to respond effectively to a social situation by inferring from indices to past actions would constitute a reproductive advantage, enabling the ‘interpreter’ to behave more adaptively (whether the signs are consciously interpreted or responded to appropriately without conscious thought); for instance: if individual A observes that B is injured and moving away submissively from individual C, A may ‘conclude’ that B lost a fight. As a consequence, A will challenge B before picking a hierarchical fight with C. Thus, the ability to interpret such indexes could develop (because the genes that encode the ability to develop the capacity to interpret such indexes would spread through the population).

In most scenarios of this class, the observer can infer a past action or constellation regardless of whether the transmitted information is reproductively beneficial to the individual observed and thus “wishes” to communicate such a past action.

To identify which cases might be relevant for language evolution, we need to introduce two distinctions concerning the information transmitted:

(1) The information could be of the type to give *the individual observed* either a reproductive advantage or a disadvantage. In situations in which understanding the past would bequeath an advantage to the observed individual, it would be beneficial for the observed individual to draw the attention of the potential interpreter to any indexes of the relevant past actions (or to hide them in the converse case).
(2) Information about the immediate past can be inferred either from a whole situation or from an object alone. While the inferences drawn from a whole situation will most often concern the immediate past and the present location (or one within sight) objects can be *carried over a distance*. Another difference between drawing inference form an entire situation versus from an object is that, regarding an object, the observed individual has more control over the communication: an object can be presented or hidden, while the inferences drawn from a situation cannot always be controlled as well.

We are particularly interested in situations in which it might be advantageous for an individual to communicate the past, because this needs to be the case to play a role in language evolution: *it must be beneficial for the speaker to communicate something* (only traits bequeathing differential fitness can be subject to evolution). The most promising scenario to be developed into a communicative situation is one in which the past can be inferred from an object and such inference gives the communicating individual a reproductive advantage.

The presentation of an indexical object could somehow be considered functionally equivalent to a linguistic utterance representing a past action; but there is also a fundamental difference: in the simplest situations in which the observer infers a past event from an indexical object, the sender need not *intend* to communicate the past action consciously.

If displaying indexical objects gave the producers of the indexes—implying their own past actions—a reproductive advantage, an index-displaying behavior would spread through the population. The system could even evolve without the presenter of the indexical object “wanting” to communicate a past action—like male Bowerbirds building a structure and decorating it with sticks and brightly colored objects to attract mates. In contrast, whenever an indexical object (including a traditional trophy of hunting or war) is presented *as a trophy*, the presenter is *trying to communicate* a past event (similarly to a modern language user who wishes to be understood).^4^ Possible precursors of this complex scenario include one in which the system would work without the presenter even wanting to be understood. Given a sufficient selective pressure, the ability of presenters to understand that audiences understand could probably subsequently develop over a long period of time. But for the development to start, neither joint attention nor the competence to attribute mental states to others (such as understanding the understanding) must be in place; rather, both could develop through use.

Regardless of whether such later developments would have been possible, the point to be made here is: *understanding* the representation of a non-present action by means of an indexical object (even if not intended by the presenter) implies a conceptual structure similar to that of the representation of a situation using a syntax-like structure: the elements of the situation must be classified in terms of thematic roles: in the case of a trophy the presenter is conceptualized as an agent and the trophy as patient, while the action *kill* is implied by the condition of the patient; thereby information about a past deed can be transmitted.

## Discussion and Some Suspicions

In this paper, we have tried to show that there is a remarkable structural and functional similarity between the cognitive foundations of linguistic syntax and the understanding of the presentation of *indexical signs* such as a skillfully made tools or hunting trophies. Hypothetically there are three possibilities:

(1) Language developed first (e.g., as inner language or for communication), after which humans could interpret presentations of indexical signs referring to the past—such as trophies—using linguistic categories.
(2) Linguistic syntax and the understanding of the presentation of an indexical sign have the same foundation (e.g., mind traveling or the ability to conceptualize) and then develop independently in the two domains. This would make them *homologous* concerning their common foundation and *analogous* in their later separate development; similar function (to refer to a non-present event) enforces similar structure.
(3) The understanding of the presentation of an indexical object was a source adaptation for language.

The claim of this paper is that regardless of which scenario is true, language evolved for its capacity to refer to past actions (and later other displaced – past, future, imaginary events –) and that this capacity could come under evolutionary pressure in various scenarios including trophy presentation. A much stronger claim (with much more exploratory power) would be to argue for the third option: inferring a past event from a present sign being a prerequisite for and progenitor of linguistic syntax, including the possibility that the presentation of an indexical object functioned as a precursor for linguistic utterances referring to the past.

In order to further consider the likelihood of this third option, it behooves us to consider more closely the types of indexical objects most likely to have played such a role in human evolution.

We have argued that one essential distinction between language and other forms of communication can be described semiotically: while non-human animals live in a world of indexes, language depends on mimetic or conventional symbols. It seems impossible to render a syntactic structure that refers to an absent action using only indexical signs. In general, indexes refer to the present, although there are exceptions, such as the traces left behind, and other examples given above. Such indexes that do refer to the past can be either complex situations or objects and the information about the agent of the indexed action can be either beneficial or adverse to the agent of the action. In the case of a display whereby comprehension is beneficial to the producer, it could be plausible for display-making behavior to evolve.

Now, we could render a menu of different possibilities: individuals may make objects that display their abilities, such as painted shells and skillfully made tools. How would objects develop during cultural transmission if they were selected for skillfulness instead of usefulness? This might be one beginning of simple forms of art. If displaying such skillfully made object would produce a selective advantage (e.g., by female choice) a *runaway process* (positive feed-back loop) might start.

Another class of indexical objects could be *corpses of animals killed in the hunt*. Differently from the skillful crafting of objects, hunting shapes the ecological conditions in which a population survives through interactions between the hunting population and their environment. Many people—e.g., Ardrey (1976)—believe that good hunters are selected according to the nutritive value of their abilities only. If this would be the case, selection would be based on the fact that families of good hunters (and thereby their genes) would flourish while other die of hunger. Selection would be mostly negative.

Hunting abilities would therefore only adapt when confronted with new environments and environmental changes. With negative selection (death by hunger) only the families of the worst hunters would be likely to die off entirely, if any; because people can survive without meat (on the other hand, high-protein food, such as meat, could definitely increase the average height and brain development of the children of good hunters). However, if the best hunters were always selected, hunting abilities could develop in a runaway-process, because competition would never end, since all hunters of the same group are competitors and hunting techniques and technology can improve with every new generation.

Also, if hunters displaying trophies were positively selected—due to social status or by sexual selection, for instance—it could become a runaway process. This might raise the value of rare trophies (such as dangerous or hard to hunt animals) over the mere nutritional value. This hypothesis is supported by the fact that when *Homo sapiens* spread around the world, the first animals to disappear were large game (Martin, 2005).

If hunting abilities evolve for the better in a population, this group will also have a competitive advantage in territorial conflicts with other groups, since hunting big game and group conflicts require similar strategies, weapons and abilities. This makes it likely that groups in which such positive feedback circles emerge would displace all other groups—who hunt merely for nutritional purposes. Because it is a good choice for a female is to mate with the most successful hunters, it is possible for the ability to interpret the value of a trophy to have evolved.

Therefore, although we wish to primarily support the more general thesis that the presentation of some sorts of indexical objects could have catalyzed the evolution of the language faculty, as precursors of utterances, we have examined the presentation of ‘trophies’ in particular as a case that most clearly demonstrates our argument.

Although our ancestors were not chimpanzees, comparing chimpanzee behavior with ours is a commonly accepted starting point for understanding the development of unique human faculties. Female chimpanzees mate more often with males that share meat with them (Gomes and Boesch, 2009). This behavior has been interpreted as sexual selection (von Heiseler, 2015). If females were to choose mates presenting an index of a rare kill over nutritional value (a choice that could be the result of runaway process that could have started, because the orientation toward rare kills produces more distinctions), they would choose mates presenting hunting trophies, giving such male conspecifics reproductive advantage.

It is also possible that the presentation of corpses of animals killed in the hunt would raise the status of the presenter among the males of the group and that the female choice would orient toward that status. This could also occur without any mental representation of past deeds: the system could work without anyone conceptualizing the events leading to the presentation of the hunted animals. A corpse of animals killed in the hunt would only turn into indexical objects when they produce mental representations of a past deed. There is another important point that concerns motivation and group structure: if trophies were to motivate actions that benefit the group, the costs that are paid by the individuals for their expensive signaling could cash out for the group.

It is possible that different groups would favor different indexical objects. Some of them could be more beneficial for the group than others. If in one group, for instance, war trophies in the form of body parts were preferred, this might give this group an advantage over groups living in the same habitat that favor rare objects and replace them slowly. Another possibility would be a skillfully manufactured object. Individuals would compete either by winning hunting or war trophies or by excelling in the artfulness of produced objects. Although both could play a role, trophy presentation seems particularly worthwhile to consider, because if then—after evolving, the tendency to choose trophy-bearers—females were to comprehend the implications of a trophy (because this should give her a reproductive advantage), this would constitute conceptualization of a past event.

Such concepts, as we have seen, would have to include categorizations similar to the thematic roles shaping syntactic structure. In this constellation, the instinct to show the trophy to a group or to a female could evolve without the intention to be understood. Because in this scenario awareness of the attentions of others can bequeath reproductive advantages, this awareness could have become subject to a selective pressure; the ability to understand how one is seen by others could have thus develop. We should consider whether a mimetic sign representing, for instance, *killing*, could have also emerged under such conditions. If such a *mimetic* gesture were to have been understood, the abilities to perform and understand such gestures could be put under a selective pressure and this might have opened the whole world of human actions to be symbolized.

## Summary

While not all indexes are of natural meaning, all signs with natural meaning can be interpreted as indexes. The idea that signs with non-natural meaning (cf. Grice, 1957) developed from indexes is therefore built on the idea that evolution often adapts what exists for new purposes. If we consider female choice a pressure in human evolution, the relevant indexical signs would somehow refer to the fitness of males. Indexes of fitness need to be selective and true, therefore they are often costly (Zahavi, 1975). We saw that one of the most essential functions of language is referring to absent actions and events. Trophies (as defined in this paper) have a similar function. At the same time, they can be costly signals. A group in which females choose trophy presenters would presumably spread through the habitat, because it would give a group an advantage in hunting, territorial conflicts, and even food gathering.

Two further reasons support the plausibility of this narrative: (1) The system could work with no comprehension (if the females simply select the trophy presenter without understanding the implications of the trophy) and that comprehension—including the imagination of a absent action—could have developed if it improved female choice. (2) The system could evolve gradually from a behavior we find in today’s chimpanzees: meat presents to females indicate the hunting ability of male chimps and make mating with the donor more likely (Gomes and Boesch, 2009).

The empirically testable prediction of this paper is: the understanding of a trophy representation involves the same brain region as the understanding of a linguistic utterance signifying the same (and therefore producing a similar imagination).

## Conclusion

In this paper, we have tried to show that there is a structural and functional similarity between the syntax of language and the presentation of an indexical object—thus we assumed that understanding the latter could constitute a precursor for understanding the former. We argued on one hand, that syntactic conceptual categories are a necessary precondition for entertaining thoughts about past actions, so that their understanding should pre-exist utterances about them. Such concepts could have developed in varieties of situations, one of which is trophy presentation (setting aside the less likely possibilities that language preceded the understanding of trophies or developed coincidentally). This is to say: we could not prove that language evolved in the context of presenting a trophy. However, this hypothesis is supported by seven arguments:

(1) A *reverse engineering argument*; the concept *to kill* implies the thematic relations *agent* and *patient* in a prototypical way (Dowty, 1991) and these could have constructed a necessary foundation for the entire range of thematic roles now used in every language of the world. With the verb *killing*, the thematic structure of agent-action-patient is most clearly pronounced.
(2) A well-known issue with language evolution—that language is too cheap for sexual selection—could be solved as follows: initially mimetic gestures would occur only as a supplement in the context of trophy-presentation (showing the killing as part of the presentation); after stories begin to circulate, the evidential function (truth indicator) could shift from the trophy itself to the circulating narratives: every story would be controlled by other witnesses and their storytelling (this will also include the development of proper names because the retelling of stories is not possible with I-narratives alone).
(3) One problem with the emergence of language (e.g., through mimetic gestures) is that it could only have evolved by improving the reproductive success of the alleles of *speakers*. This would probably be the case if the first mimetic gesture developed in the context of trophy presentation.
(4) *Problem of altruism.* The (direct or indirect) sexual selection of presenters of trophies could explain why altruistic behavior may be *evolutionarily stable*. Once narrations circulated within populations and the abilities emerged to attribute respect and disrespect to individuals depending on the stories circulating about them, a new social structure would have developed—what we would like to call *narrative altruism*. In this case altruism would be a result of language evolution, in contrast to theories that altruism was a necessary condition for the development of language.
(5) *The paradox of the first linguistic utterance*: language (as understood in this paper) needs a minimal complexity: argument structure (an n-valent verb + n arguments). Such a structure seems too complex to be produced spontaneously and understood instinctively within a communicative act. There are two common suggestions to solve this problem: some researchers believe holistic protolanguage to be the precursor of language; others believe that words emerge first and later syntax. Both concepts are problematic: (a) Holistic expressions [like drumming on a tree trunk, for instance, as a chimpanzee male may do to indicate a hunting trip is to begin (Stanford, 1999), interpreted as the holistic expression “Let’s go hunting”] are not linguistic utterances, but a common part of animal communication. (b) On the other hand, a sign that refers to an object [e.g., a warning call that distinguishes between eagle, snake, and feline predator (Seyfarth et al., 1980)] could emerge; but how syntax emerge from such calls? In the common view there cannot be any syntax without words. But aren’t words—differently from other signals—only words because they can participate in syntactic structures? If trophy comprehension preceded linguistic utterances, this paradox could be solved: a syntax-like structure could emerge without words (presenter = agent, trophy = patient, verb implied in the state of the patient). As a second step a first ‘word’ (verb) in the form of a mimetic gesture could have emerged signifying the *killing*.
(6) *Overcoming the problem of circularity*. Logically, a behavior cannot be performed without the ability to perform it. However, in the evolutionary process, this relation between behavior and the ability to perform a certain behavior is inverted. The justification for this *strange inversion of reasoning* (Dennett, 2009) is that a behavior (with selective advantage) is the cause of (the development) of the ability to perform this behavior. This paradox can be unfolded in the evolutionary process: an action can be performed in a primitive way and maybe even by chance. If this performance increases the reproduction of the allele of the individual, the genetic foundation of the ability can improve over generations by natural or sexual selection. For this process to happen, we need two conditions: (a) the behavior in question needs to generate a reproductive advantage and (b) among the individuals of a population there must be variations of the ability to perform the behavior resulting in variation of reproductive success (fitness). In the case of comprehension (a) a simple—maybe unconscious—form of a behavior emerges, (b) the reproductive advantage of the behavior can be improved by comprehension. In our example, in its simplest form, females choose males that retrieve trophies without interpreting the trophies as trophies (and thereby as information about an absent action). The development could start with a behavior we see with Chimpanzees: males hunt and share some of the meat with some females (Gomes and Boesch, 2009). The females are then more likely to mate with them. If we interpret this as a form of sexual selection (evidence for this is that females – though they are sometimes present – do not join the hunt)^5^, the signal could get more and more selective, becoming the index of a rare kill. This structure could develop toward understanding of the implications of the index (trophy), including the imagination of a past action. This would suggest that the conceptualizing of a past action would first be selected in females and spread from them in the next generation to both sexes as is in general the case when the trait is not a disadvantage for either sex.
(7) Sexual selection by one sex has at least two conditions: the freedom of choice of the sexual partner and a difference in the parental investment between sexes. The cost difference in parental investment between the sexes correlates with the choosiness of the selecting sex [in most cases the females (Bateman, 1948)] and the choosiness of the selecting sex correlates with the cost of the courtship behavior of the chosen sex (Trivers, 1972). This is to say: the differences in parental investment corresponds with the cost of the courtship behavior of the selected sex (in most cases the males). As the total amount of parental care would have grown along with the increasingly earlier births of our ancestors, the costs of the courtship behavior for the males should grow and the females should become more and more discriminating (and the behavioral dimorphism get accordingly more expressed). In this situation the reproductive success of females relies heavily on the best mating partner choice, because the best strategy to pass their genes onto the next generation is to combine them with good genes. Non-human animals make their choices based mainly on the male’s social status and perceptible appearance. If, females develop comprehension of the implications of indexes such as trophies, they can improve their mating choice competence. This would even make small differences in the comprehension of indexical signs evolutionary relevant.

To defend the presumably controversial hypothesis that language actually evolved from trophy presentation, there are good reasons for narrowing down the time corridor for when this development could have happened (though this paper was not about dating when language evolved). One reason would be: we have assumed some elements of social structure and a behavioral sexual dimorphism among our ancestors. For this structure to be verifiable by empirical data we need to know what time period we are talking about. Obviously, we are not discussion contemporary populations of hunter-gatherers. In these societies language is in place and as a result stories about individuals circulate. These circulating narratives store and distribute the reputation of the individuals. This makes certain forms of cooperation and costly (apparent altruistic) behavior likely (von Heiseler, 2015).

It was suggested that the exceptional brain growth of the Homo erectus seems to have been associated with the development of uniquely human cognitive faculties, including simple language (Everett, 2017). Now, if trophy presentation was prior to linguistic communication it must have occurred even earlier. Because developments often start rather slowly it is likely that between the first meat presents to females, through the instinctive tendency to present trophies, and the slow dawning of the imagination of past actions, to the full understanding of indexical signs of displaced actions, could have taken millions of years.

Our approach corresponds to the *function-first* idea: something (whatever exists and can be used) is used to fulfill a function (suggesting a past action) which causes selective pressure to further develop that function.

Obviously, we have not presented a complete scenario for language evolution here. Such a theory would have much else to explain and to explain the ‘trophy’ selection scenario in greater detail. Here we have tried to only establish the unique role indexical signs are likely to have played in language evolution, and to suggest that trophy selection provides an ideal exemplar of the principle.

## Author Contributions

The author confirms being the sole contributor of this work and has approved it for publication.

## Conflict of Interest Statement

The author declares that the research was conducted in the absence of any commercial or financial relationships that could be construed as a potential conflict of interest.

## Footnotes

1 In this paper we discus syntax mainly in its simplest from. This is a verb with (necessary) arguments and excludes adjuncts, embedding and complex hierarchal structures. The development from minimal syntax to complex syntax is not the topic of this paper. We are aware that this view contradicts Chomsky’s notion that there cannot be any syntax without hierarchy and recursion.

2 The first letter of the Phoenician alphabet is revealing for two reasons. Firstly, on one hand, it is conventional in the sense that nobody will see a bull head in the sign without knowing about it, but on the other hand, anyone can see the icon of the bull-head in it once described. Secondly, because the production of the sign (drawing it) influenced the development; the line from upper-left to lower-right is longer than would be expected.

3 *Arbitrarisation* can be a fascinating topic and a model for describing *language change* (e.g., from Old to Modern English). Impressive work has been done by Luc Steels, who let Sony-sponsored little robots communicate until they equalized their signals for some colors and objects; and by researchers from the University of Edinburgh, who showed that conventionalization can also be influenced by the limitations and constraints of human memory (Cornish et al., 2017). However, neither has anything to do with the *origin* of language.

4 We define *trophy* as an object implying that the presenter performed an action that requires exceptional abilities and personal qualities—in the case of hunting trophies, exceptional hunting abilities, strength, and courage. Thus, a trophy is an object, which communicates something about the presenter of the trophy that is beneficial for him to communicate. The cost of acquiring such a trophy might include putting oneself in jeopardy. In this case it is a form of expensive signaling.

5 Behavioral sexual dimorphism is often an index for sexual selection.
